# Fifteen years with patient choice and free establishment in Swedish primary healthcare: what do we know?

**DOI:** 10.1177/14034948221095365

**Published:** 2022-05-20

**Authors:** Mio Fredriksson, David Isaksson

**Affiliations:** Department of Public Health and Caring Sciences, Uppsala University, Uppsala, Sweden

**Keywords:** Patient choice, scoping review, primary healthcare, primary care, any willing provider laws, choice of healthcare provider

## Abstract

**Background:**

In 2007, a reform of Swedish primary healthcare began when some regions implemented enhanced patient choice in combination with free establishment for private providers. Although heavily debated, in 2010 it became mandatory for all regions to implement this choice system.

**Aim:**

The aim of this article was to review all published research articles related to the primary healthcare choice reform in Sweden, to investigate what has been published about the reform and summarise its first 15 years.

**Methods:**

A scoping review was performed to cover the breadth of research on the reform. Searches were made in Scopus, Web of Science and PubMed for articles published between 2007 and 2021, resulting in 217 unique articles. In total, 52 articles were included.

**Results:**

The articles were summarised and presented in relation to six overarching themes: arguments about the primary healthcare choice reform; governance and financial reimbursements; choice of provider and use of information; effects on equity and access; effects on quality; and differences between private and public primary healthcare centres.

**Conclusions:**

**The articles show that the reform has led to an increase in access to primary healthcare, but most studies indicate that the increase is inequitably distributed in terms of socioeconomy and geographical location. The effects on quality are unclear but several studies show that the mechanisms supposed to lead to quality improvements do not work as intended. Furthermore, from a population health perspective, it is time to discuss how such a responsibility can be reintegrated into primary healthcare and function with the choice system.**

## Background

Inspired by new public management, there has been an increasing focus on patient choice in Northern Europe and the Nordic countries since the beginning of the 1990s [1, 2]. In Denmark and Norway, patient choice policies have mainly targeted specialised care [3, 4]. This also applied to Sweden up until 2007, when some of the 21 regions – which are responsible for funding and providing healthcare – started implementing reforms enhancing patient choice in combination with privatisation in primary healthcare (PHC) [5, 6]. In 2010, the PHC choice reform made this type of choice system mandatory for the regions. The purpose was twofold: to strengthen patients’ choice of provider and to make it easier for private providers to establish a PHC centre with public reimbursement [7]. Many regions already provided the opportunity to choose PHC centre but importantly the reform added freedom of establishment for private providers that fulfilled the requirements determined by each region and a ‘money follows the patient principle’. Similar changes have been suggested but not implemented in Finland [8].

The political majority behind the reform claimed that a diversity of providers – which would compete with each other on the same terms for patients to get reimbursed – would enhance cost efficiency, improve access and stimulate quality improvements. Access and quality would improve because many patients would choose the provider with the highest quality and best access [7]. Importantly, the reform meant that private and public PHC centres now operate in the same market, on the same terms and are reimbursed according to the same principles within a region. There are regional variations in the reimbursement systems although all are foremost based on capitation with a risk-adjustment component that is, for example, related to socioeconomic status, registered diagnoses or remote location of a PHC centre. The Health and Medical Services Act (2017: 30) applies to both public and private providers and establishes that PHC refers to outpatient care without limitations in terms of diseases, age or patient groups. Its focus is medical assessments, treatments, care, preventive work and rehabilitation that do not require special medical or technical resources or any other special skills.

The reform in 2010 meant a sharp break with the traditional Swedish PHC model, which is rather unusual in an international perspective. The traditional model centred on publicly owned PHC centres (70% before the PHC choice reform [9]) with responsibility for population health within a geographical area (*områdesansvar*), employing a multidisciplinary workforce, typically with four to 10 general practitioners (GPs) and other professionals such as district nurses, physiotherapists, social workers, work therapists, psychologists and specialist nurses [5]. With the reform, from 2010 the PHC centres’ responsibility for population health within a geographical area disappeared [10]. According to Anell [11], the PHC choice reform meant that Sweden now ‘has more liberal rules for market entry and private ownership of primary care providers than do many European countries with long traditions of regulated private markets’. However, although a larger proportion of PHC centres now are owned by private companies (44%) (Isaksson D. *Data compiled from the regions*. Unpublished, 2022), the Swedish PHC model with PHC centres with a multidisciplinary workforce still remains. As before, the patients also make co-payments for a PHC visit (between SEK 150–300), up to the sum of SEK 1200 per year in 2022.

At the time of the introduction of the reform in 2010, there was a heated societal, professional and political debate on what the effects would be for PHC, in particular discussing potential effects on geographical equity and distribution of PHC based on socioeconomic conditions. Since 2010, studies investigating different aspects of the PHC choice reform have been published, but no comprehensive review of these studies has been conducted to summarise what we know about the functioning and effects of the reform. We argue that it is important to summarise the existing evidence because the reform is unique in a Nordic context due to its combination of patient choice and free establishment for providers. It is also important to inform policymaking on PHC in an era of increases in long-term conditions and financial pressures on healthcare systems.

## Aim

The aim of this article was to review all published research articles related to the PHC choice reform in Sweden, to investigate what has been published about the reform and summarise its first 15 years.

## Methods

We chose to conduct a scoping review because it is appropriate when the topic to be covered is broader compared to a systematic review, when different study designs are anticipated, and the studies will not be quality assessed. A scoping review may be useful to map a research field regarding, for example, the extent, range and nature of research activity and to summarise and disseminate research findings. The review was carried out in accordance with the five obligatory stages of a scoping review of Arksey and O’Malley [12]: (a) identifying the research question; (b) identifying relevant studies; (c) study selection; (d) charting the data; and (e) collating, summarising and reporting the results.

We identified the research question to be: What do we know about the functioning and effects of the PHC choice reform? The relevant studies were identified through a literature search in three databases: Scopus, PubMed and Web of Science. A first search was conducted on 20 August 2021 and a complementary search was conducted on 20 January 2022. The search terms (Sweden OR Swedish) AND (choice OR ‘free establishment’ OR reimbursement OR incentives OR privati[s/z]ation OR marketi[s/z]ation) AND (‘primary care’ OR ‘primary healthcare’ OR ‘primary health’). We included peer-reviewed studies published in the English language between 2007 and 2021. The year 2007 was chosen as a starting point because that was the first year a Swedish region introduced a PHC choice system. We also hand-searched the reference lists in the articles included in the final review.

**Figure 1.**
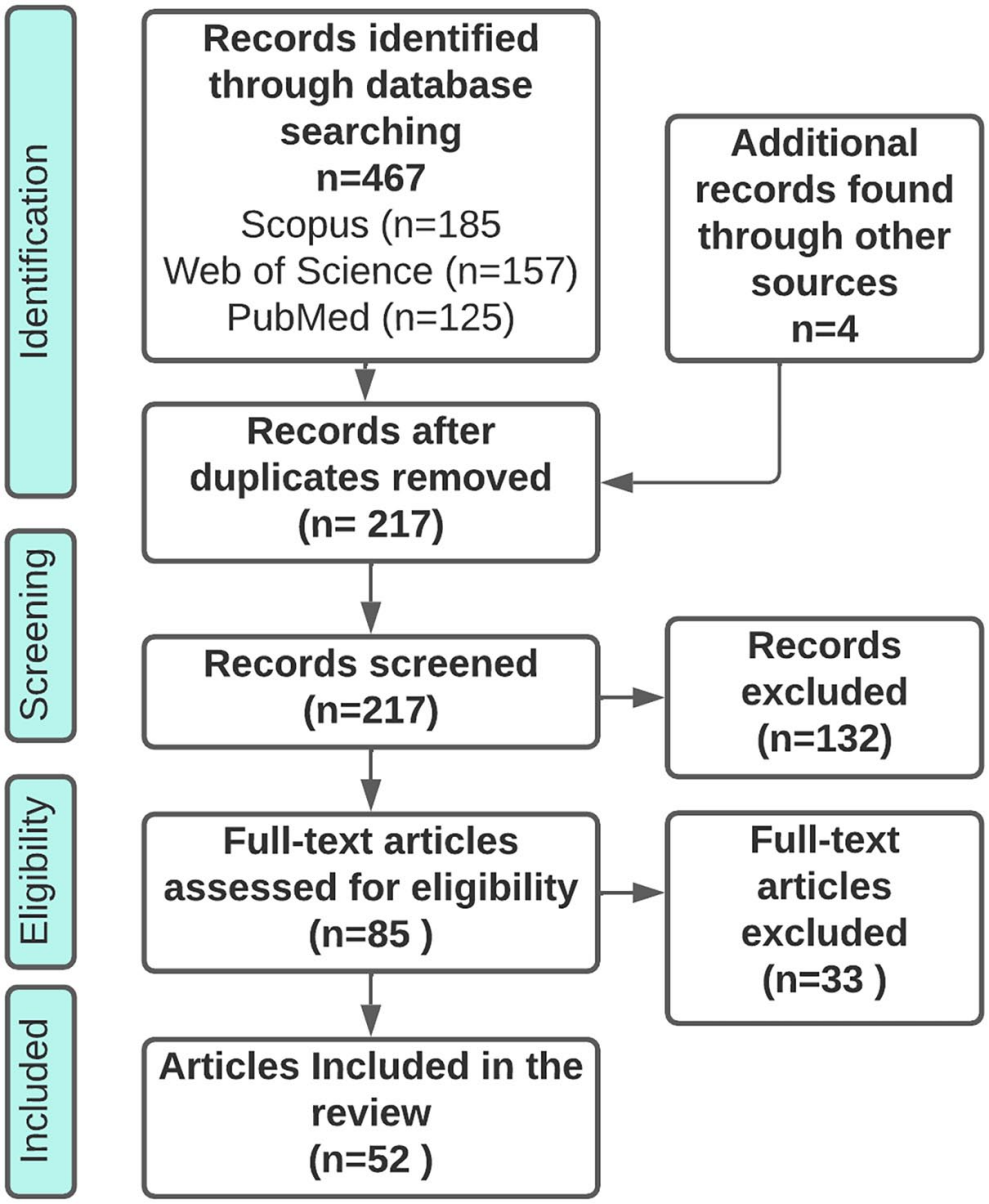
Flowchart of the review process including database searches, number of abstracts screened and full text articles retrieved.

Studies were selected by first merging the three database searches (125 studies were identified in PubMed, 185 in Scopus and 157 in Web of Science) and then removing duplicates manually: resulting in 213 unique articles. In addition, four studies were found through a screening of the references of the included articles. Thereafter, both authors read the title and abstract of these articles together to assess whether they should be read in full text: resulting in 85 articles. The authors read half of these articles each and in discussion with each other decided that 52 articles met the inclusion criteria see Figure 1. The included articles were entered into a data-charting form in which information on title, authors, design, geographical scope and theme was recorded, see Supplemental material. We included articles that discussed the PHC choice reform or illustrated how the PHC choice reform has been implemented, has functioned, been governed and what effects it has had on a range of aspects, for example, access, equity and quality. We included qualitative and quantitative articles as well as theoretical and empirical articles. Articles that studied aspects of Swedish PHC without mentioning the PHC choice reform were excluded. The results are summarised and presented in relation to six overarching themes of relevance to the functioning and intended effects of the reform: the arguments about the PHC choice reform; governance and financial reimbursements; choice of provider and use of information; effects on equity and access; effects on quality; and differences between private and public PHC centres.

## Results

Of the 52 included articles, 29 were quantitative, 20 were qualitative, two utilised a mixed method and one was a review article. Twenty of the included articles had a geographical scope that covered the entire country, whereas 26 articles covered between one and three regions, see Table I and the Supplemental material for more information about the included articles.

**Table I.** Overview of included articles.

Theme	No. of articles	Study topics
Arguments about likely effects	6	Possible effects on equity, governance, working conditions and quality
Governance and reimbursement systems	20	How PHC is governed after the reform, reimbursement incentives and effects of reimbursement systems
Choice of PHC centre and use of information	13	Opinions on choice, search and use of information, availability of information, important factors when making a choice, information on patient exits
Effects on access and equity	7	Number of PHC centres, number of GP visits per capita, distribution of visits among patient groups and location of PHC centres
Effects on quality	3	Patient satisfaction and objective quality measures
Differences between private and public PHC centres	3	Prescription patterns and perceived quality

GP: general practitioner; PHC: primary healthcare.

### Arguments about likely effects

Before the full introduction of the PHC choice reform in 2010, potential effects were discussed by researchers, predominantly by those who warned against negative effects. Fears were voiced that existing social inequities in access would be reinforced [13–15]; that it would benefit the healthier and more well educated to a larger degree [15]; reduce the opportunities for politicians to steer towards equity (i.e. needs-based geographical resource allocation) [13–15]; increase public expenditures for PHC [13, 14] and lead to a fragmented healthcare system [13, 16, 17]. On the positive side, the potential for improved working conditions for GPs was mentioned [15]. There was also a critique against a lack of evidence of the anticipated effects of the reform [14] and Fredriksson et al. [18] illustrated the lack of estimations of the effects on equity in the preparatory work.

### Governance and reimbursement systems

Regarding the overall governability, in 2015 Saltman and Duran [19] noted that the PHC choice reform had meant that substantial areas of decision-making left public hands, for example, where to locate new PHC centres (also mentioned by Anell [11] and Kullberg et al. [20]) and whether to allow private for-profit firms into PHC. Similarly, Fredriksson [21] illustrated that while the PHC choice reform empowered individual patients, it simultaneously weakened the collective voice of citizens because their elected representatives could no longer control the number or location of PHC centres.

In 2019, Glenngård [22] showed that all regions now use four types of control measures to steer PHC: contracts; reimbursement systems; dialogue; and performance measurement systems (mostly structure and process measures). Regional representatives regarded dialogue as most important, as it builds trust, relationships and shared knowledge between the region and the PHC providers. In 2015, Norén and Ranerup [23] found that the contracts in two studied regions included quality indicators which were followed up and made public, but concluded that there were no measures of clinical quality or outcomes. Linked to this, Arvidsson et al. [24] found that the regions audited non-clinical measures such as utilisation of resources and the volume of production, and thus checked on the fulfilment of contractual obligations rather than spurring quality improvement. However, using data on the volume of visits and patients’ judgement about the quality of services from 2010, Glenngård [25] found no conflict between productivity and patient satisfaction.

In the study by Glenngård from 2019 [22], the reimbursement system was pointed out as potentially the most powerful control measure. A year after the full introduction of the PHC choice reform, Anell [5] noted that a majority of the regions had chosen capitation reimbursement to PHC providers and a comprehensive responsibility for the PHCs (for up-dated descriptions of reimbursement systems, see Anell et al. [6] and Winblad et al. [26]). The new financial incentives after 2010 made managers of publicly owned PHC centres note a shift in power towards the patients, improving access and service; however, also leading to more patients with unreasonable demands [27]. The financial incentives also led to innovations such as the opening of drop-in units, changed opening hours and the adding of new business to the PHC providers’ core activities, fore example, a collaboration with a fitness centre and with companies offering life insurance [28]. Similarly, Vengberg et al. [29] showed that both managers and GPs at PHC centres were highly aware of the financial incentives and that fee-for-service reimbursements were perceived to increase the production of shorter visits and skimming of healthier patients. The adjustment of reimbursements based on diagnoses also led to an increased focus on registering diagnosis codes, which in some cases could lead to ‘upcoding’ of secondary diagnoses.

The importance of the reimbursement systems was also illustrated by Winblad et al. [26] in a study investigating whether the regions employ strategies to avoid unwanted risk selection by providers. The authors found three main strategies: risk adjustment of the financial reimbursements on the basis of health and/or socioeconomic status of listed patients; the design of patient listing systems; and regulatory requirements expressed in the contracts regarding the scope and content of the services. Focusing in particular on rural regions, Kullberg et al. [20] showed that the PHC choice reform undermined resource-allocation systems based on health needs and undercut attempts by local policymakers to plan for care provision in remote locations. The authors concluded that competition is not suited for the provision of healthcare in rural areas, but also noted that negative effects can be moderated through financial reimbursements. One example of a socioeconomic adjustment to capitation reimbursements currently used in all Swedish regions is the care need index (CNI). Anell et al. [30] examined the association between the CNI and PHC visits in three Swedish regions. The results showed a small correlation between PHC centres with a high CNI (low socioeconomic status) and the number of GP visits. The correlation could, however, be explained by a few PHC centres with very high CNI scores. The authors concluded that the results may indicate insufficient compensation based on the CNI or that the extra reimbursement based on the CNI is spent on aspects other than GP visits.

Several articles examine the effects of different reimbursement models related to the PHC choice reform. How diagnosis coding changed after an introduction of economic compensation based on the reported diagnoses was investigated in two articles. In 2012, Hjerpe et al. [31] showed that hypertension diagnoses increased from 17.4% to 32.2% while cancer diagnoses increased from 0.79% to 2.32%. Similarly, Dackehag and Ellegård [32] found a general increase in the number of registered diagnoses after the introduction of a reimbursement system that adjusted the reimbursement based on registered diagnoses. Their results also showed that PHC centres in competitive markets increased their number of registered diagnoses by ~3% more than PHC centres in less competitive markets.

Pay-for-performance (P4P) reimbursements in the Swedish PHC choice system is the topic of a few studies. In 2015, Ödesjö et al. [33] showed that in a region with P4P reimbursements related to diabetes programmes there was an increase in patients with a blood pressure just below the target values, but not in a reference region that had not implemented the P4P programme. The authors concluded that this indicated that P4P can lead to ‘gaming of the system’. In contrast, Ellegård et al. [34] showed that the use of a P4P reimbursement related to the type of antibiotics being prescribed when treating children with respiratory tract infections increased the share of narrow-spectrum antibiotics. The authors did not find any indications of gaming by prescribing more antibiotics overall to increase the share of narrow-spectrum antibiotics. Furthermore, Ellegård [35] examined the effect of P4P on compliance with hypertension drug guidelines among public and private healthcare providers. The results showed that P4P incentives had a significant effect on guideline compliance and that the effect was most noticeable for private providers, indicating that private providers are more responsive towards financial incentives.

### Choice of PHC centre and use of information

In 2007, when only a few regions had introduced PHC choice systems, Hjelmgren and Anell [36] found that, in a hypothetical choice of PHC provider, short waiting times and the level of influence over the care received were more important than the possibility of choosing a specific GP or low user charges. A few years later, a survey in three regions that were early adopters of the PHC choice reform showed that as many as 70% were strongly in favour of having the possibility of choosing a PHC provider (similar to Ranerup et al. [37]) and that about 60% of the population felt that they had made a choice of provider (often one they had previously been in contact with) [38]. The likelihood of making a choice of provider increased almost three times if people said they had enough information to be able to make a choice.

The study thus pointed to the importance of information when making a choice of provider (a supply-side factor). To what extent such information exists was investigated by Ranerup et al. [39] 2 years after the PHC choice reform. The authors found many differences between four web portals regarding what types of data were presented. No web portal provided a function for sorting and ranking different options, thus reducing the possibility of making informed choices. In another study, Ranerup et al. [37] concluded that of the existing information, people rated information on opening hours location and waiting times most important.

Some studies focused on how individuals search for and use information when making a choice of PHC provider (demand-side factors). They all indicated that information is not used to a large extent. For example, Wahlstedt and Ekman [40] showed that the use of Internet-based information sources was relatively low in 2010 and only slightly higher in 2013 (5.8% and 6.4%, respectively). Similarly, Hoffstedt and colleagues reported, in two studies, that in actual choice situations, patients searched for information to a limited extent [41, 42]. Only 17% responded that they had searched for information to a very large or large extent [41]. The type of information patients searched for was of more basic character (e.g. localisation of the PHC centre) and came from potentially biased sources such as the PHC centre they wanted to switch to or family and acquaintances [42]. The strategy of asking family and acquaintances illustrates that recommendations from other PHC users are important. Abrahamsson et al. [43] found that treatment encounter responsiveness (being listened to, getting understandable information, being respected, etc.) was strongly associated with recommending a provider. Similarly, the overall impression of the PHC visit was used as a measure of subjective quality in the study of Dahlgren et al. [44] of actual choices of provider, together with avoidable hospitalisation as a measure of objective medical quality. The study showed that distance was the most important factor in choosing a PHC centre and that the willingness to trade distance for quality was marginal. A previous interview study also pointed to the importance of distance and waiting times when choosing a PHC provider, but of continuity of care as well [45].

There is also some evidence in the literature about who searches for and uses information. Those using Internet-based information sources were younger, more educated, more often women and had better self-perceived health [40]. Furthermore, Hoffstedt et al. [41] demonstrated that not only aspects such as gender, educational attainment and employment status were associated with the level of information-seeking when switching provider, but also motivation. Those motivated by internal factors such as dissatisfaction or a belief that another PHC provider may provide better services actively sought out information to a greater extent. In line with this, in a field experiment sending comparative information regarding PHC centres to patients by mail, Anell et al. [46] showed that it increased the likelihood of a patient switching PHC centre by 10–14%. The authors interpreted this as evidence of demand-side friction in the PHC market, and that patient mobility can be supported with accessible information. Little is currently known about how many switch PHC centre because they are dissatisfied. The experience of having switched PHC centre when dissatisfied has, however, been found to correlate with the support of further privatisation of healthcare [47]. Furthermore, in an interview study with managers and physicians at PHC centres, Vengberg et al. [48] found that the providers lacked information on patients’ choices, and when (and why) patients exit. The authors concluded that the lack of information makes it difficult for the providers to respond to patients’ choices by adapting their services and that it is questionable whether choice and competition stimulate enhanced clinical quality.

### Effects on access and equity

Regarding access, Isaksson et al. [9] showed that the number of PHC centres increased from 1089 before the reform to 1374 in the year 2013, an increase of 26%. How equity has been affected by the PHC choice reform is investigated in a handful of studies. In 2017, Burström et al. [49] conducted a review (including grey literature) evaluating the equity impact and concluded that the PHC choice reform may have damaged the equity of PHC provision.

Three studies have investigated GP visits. Analysing register data from the years 2007–2011 in Region Skåne, Beckman and Anell [50] showed that healthcare utilisation increased overall, both measured as the number of individuals visiting a GP and the number of GP visits per capita. Furthermore, the results showed that utilisation increased most among individuals aged 64 years and above with a higher household income than the average. Using data from 2006–2010 in Region Stockholm, Agerholm et al. [51] found that, although there was a general increase in visits to GPs following the reform, the increase was smaller among patients with poor mental health and longstanding illness. Furthermore, 8 years after the PHC choice became mandatory, Sveréus et al. [52] analysed the socioeconomic distribution of GP visits in the three largest Swedish regions using data from 2 years before the introduction of the PHC choice system and 2 years after. The results showed that the reform led to a general increase in utilisation of PHC but that there were only small changes in the socioeconomic distribution. Over time, utilisation increased in lower income groups.

Furthermore, Isaksson et al. [9] found that new private PHC centres in Sweden were, in general, located in more affluent areas compared to public PHC centres. This may indicate that the reform led to a decrease in geographical equity because more affluent areas got more new providers. In line with this, another study by Isaksson et al. [53] analysed socioeconomic differences in patients registered with private compared to public PHC centres. The results confirmed that individuals with higher socioeconomic status were more likely to be registered with a private PHC centre than individuals with lower socioeconomic status. Studying only Region Skåne, Lindström et al. [54] compared the perceived unmet healthcare needs between patients registered with private compared to public PHC centres while controlling for socioeconomic status and self-rated health. The results showed no statistically significant differences between the groups.

### Effects on quality

Two published articles aimed to evaluate the impact of the PHC choice reform on different types of healthcare quality. In 2020, Dietrichson et al. [55] found small improvements in subjective quality, that is, patients’ satisfaction with care, but no clear effects on objective quality measures such as avoidable hospitalisation. However, a study by Glenngård [56] indicated that patient satisfaction with PHC centres is dependent on structural and organisational characteristics such as the size of the PHC centre and composition of registered patients. As these factors are difficult for a PHC provider to influence, it might not be optimal to use patient satisfaction to assess PHC quality.

Furthermore, in an ecological register study from 2021, Mosquera et al. [57] evaluated how the PHC choice reform has impacted emergency department visits and hospitalisations in all Swedish regions. The authors found only a small possible effect of the reform and concluded that the study does not provide any evidence of improved performance of the PHC system in Sweden as an effect of the PHC choice reform.

### Differences between private and public PHC centres

A few studies have investigated differences between private and public PHC centres, of which three studies investigated prescription patterns and/or perceived quality. The prescription of antibiotics was investigated by Maun et al. [58] and Granlund and Zykova [59]. In the study by Maun et al. [58], the authors found a higher prescription rate of antibiotics for private PHC centres (6.0 vs. 5.1 prescriptions per 100 individuals in a quarter) but a lower prescription rate of benzodiazepines. Due to a lack of case-mix the authors could not rule out that the differences could be explained by differences in patient characteristics. However, the same pattern was found by Granlund and Zykova [59] in a register study from one region. The results showed that physicians employed at private PHC centres were 6% more likely to prescribe antibiotics and 9% more likely to prescribe broad-spectrum antibiotics compared to physicians employed at public PHC centres.

The study by Maun et al. [58] also investigated perceived patient quality. The authors found that private PHC centres had a higher perceived quality compared to public PHC centres (82.4% had a high perceived quality compared to 79.6%, although not case-mix adjusted). The same trend was spotted in a recent study in which the results showed that private PHC centres had a higher perceived quality [60]. Among the private PHC centres, the perceived quality was higher for smaller independently owned PHC centres compared to PHC centres owned by larger corporate groups. No case-mix was used in the analysis.

## Discussion

The Swedish PHC choice reform was intended to strengthen patients’ choice of provider and facilitate the establishment of private PHC centres. The idea was that a diversity of providers competing for patients and the accompanying public reimbursement would enhance cost efficiency, improve access and stimulate quality improvements. Critics, however, argued that the reform could impair equity because the regions would no longer be able to steer the location of PHC centres directly to areas with higher care needs, and because not everyone has the capacity to make informed choices. The review of published studies may help us answer whether the anticipated effects have been realised and to understand how the PHC choice reform’s mechanisms function, how PHC is governed and how PHC centres respond to different types of financial incentives. Overall, the results are consistent with previous research on patient choice, which has, however, mainly focused on hospital choice [61–63].

With regard to governance, the reviewed studies suggest that the regions lost some of their governing capacity, that is, their traditional way of governing by way of planning and budgets. However, the transfer of power to the patients, who, with their choice of provider, allocate resources, was intentional and the studies show that the regions now use other governing and control measures such as financial reimbursements, dialogues and contracts. Furthermore, the regions try to regulate the PHC market to meet important values such as geographical equity and provider quality (e.g. through risk-adjusted reimbursements), but there are questions about how effective this type of regulation is. Several studies suggest that steering via clinical quality measures is underdeveloped (a type of steering the professionals support), which is likely to be linked to the lack of systematically collected data on PHC quality in Sweden. Furthermore, the reviewed studies conclude that financial incentives are powerful and affect both the actions of PHC centres and professionals. All PHC centres respond to financial incentives but it seems as if private PHC centres respond to a greater degree.

Regarding patients’ choices, the review suggests that people want the possibility to choose PHC provider. In addition, it seems as if having enough information is associated with the likelihood of making a choice, but at the same time, the reviewed studies indicate that people search for information about providers to a limited extent. In particular, it is rare that people search for information about the providers’ quality and they more often ask family and friends for advice (which has also been established internationally) [61]. However, to what extent the low degree of information-searching reflects a lack of easily accessible information about providers and their quality is unclear. At present, there is no platform that allows easy comparison of PHC centres on the basis of objective quality measures (a supply-side barrier). Thus, it is questionable whether people can act as informed consumers and, in addition, Victoor et al. [61] have shown that the choice process is much more complex than often assumed and that many patients are unable and/or unwilling to make completely rational choices of provider. The latter is an assumption underlying the PHC choice reform: that informed choices will lead to better quality because patients will choose providers of higher quality, thus providing them with the means to expand their practice while PHC centres of low quality will either have to improve or close down. Thus, switching provider when not satisfied is an important mechanism for the PHC choice system to work as intended [25]. However, information on how many Swedes that exit a provider due to dissatisfaction with services or a belief that another PHC centre can offer better quality is not available. Most previous research on patient choice suggests that proximity is the most important factor when making a choice and that ill patients want to rely on a trusted practitioner [63], indicating a barrier for exit. Interestingly, information about patient exits is also not easily available to the PHC centres, which thus cannot improve their services to meet patients’ needs and expectations. However, it is possible that the PHC centres get some other type of feedback from patients, for instance through e-mails or telephone calls to the manager. An important thing to note is that exit is only possible when there are alternatives to choose from and the exit mechanism therefore does not fully work in rural areas.

Taken together, the studies of the effects of the PHC choice reform on access and equity show that access has increased, both the number of PHC centres and utilisation. It is, however, uncertain how these increases in access are distributed among different patient groups because the reviewed studies show somewhat contradictory results. Most studies, however, indicate that the PHC choice reform has had a negative impact on equity and that the increase in utilisation is greater among individuals with higher socioeconomic status, which is consistent with previous research [62]. However, there is a lack of information on the length and content of PHC visits, making it precarious to draw any firm conclusions on the effects on access and equity. Nonetheless, it is clear that private PHC centres, established after the PHC choice reform was introduced, are found foremost in more prosperous areas and that patients with higher socioeconomic status are more likely to be registered with a private PHC centre. Patients also seem more satisfied with private PHC centres, which according to two studies have higher perceived quality, but two studies also show that private PHC centres prescribe more antibiotics. However, the effects of the PHC choice reform on quality are still understudied and the few published studies did not find any clear direction of the results. It is also notable that we found no published articles focusing on how the reform has affected costs, efficiency and continuity. A lack of evidence of improved efficiency and quality as a result of patient choice has previously been noted [63], but some studies have indicated a positive effect of competition on quality in specialised care [64, 65].

To sum up its first 15 years, we can conclude the PHC choice reform has, to some extent, had the intended effects. Access has increased both in terms of utilisation and the number of PHC centres, and patients’ choice has been strengthened. In addition, some of the critique against the reform has been shown to be justified, in particular with regard to equity, in which several studies indicate that the increase in access foremost has benefitted socioeconomically stronger individuals. Regarding the reform’s effect on quality, the evidence is unclear but several studies show that the mechanisms supposed to lead to quality improvements do not work as intended. One problem is the lack of clinical quality information to guide patients’ choices, and another, that providers do not know when a patient has used the ‘exit’ mechanism. Furthermore, it is unclear whether popular providers are able or willing to expand. The high costs of expanding a PHC centre by acquiring new facilities and staff may limit the economic incentives to expand even for providers with high demand [55], and some may feel that an expansion would damage their ability to provide high quality care.

Another intended effect is that the PHC choice reform increased the number of private PHC centres. The studies indicate that private providers are more susceptible to financial incentives (even though public providers also respond strongly to financial incentives). Thus, the reimbursement models become a key component in steering PHC and can create positive as well as negative outcomes depending on their design. Policymakers must therefore analyse potential effects and be ready to change the reimbursement models if they find that the incentives lead to unwanted effects. What policymakers must also do is to support the development of PHC quality information that can be used in the choice of provider, and ensure the development of a tool or platform that can be used to compare PHC providers based on relevant quality indicators, and that supports those with reduced capacity to make a choice of provider.

It is important to note that although this review focused on a patient choice reform, the studies rather illustrate different aspects of the patient choice system in Sweden, which also incorporates free establishment (any willing provider). This means that a company that fulfils the requirements set up by a region is allowed to establish a PHC centre anywhere within the region, even in an area where there is an overprovision of PHC. Further regulation of providers and their establishments has been suggested, for example, making it possible for the regions to have different rules in areas with difficulties attracting providers (SOU 2019:42), and the abolition of the PHC choice system for older people with great care needs (SOU 2016:2). However, the challenges in Swedish PHC go beyond the PHC choice system (SOU 2019:42); for example, the general undercapacity in PHC (Sweden spends below the EU average on PHC as a share of total health spending) [66]. Overall, a strengthening of PHC is taking place within a comprehensive reorientation of the Swedish healthcare system toward close provision of care (*God och nära vård*). It has also been argued that the PHC providers’ responsibility for population health within an area should be reintroduced. A commission suggested in 2017 (SOU 2017:47) that providers should engage in preventive and health promotion efforts in collaboration with civil society and municipalities. This reasoning is in line with the World Health Organization’s approach to PHC as ‘a whole-of-society approach’ and international calls for integrating PHC and public health [67, 68]. Potentially such a development can come with the transition to *God och nära vård*.

Finally, there are a few limitations to this review study. It is possible that we missed relevant studies, in particular on the effects on clinical quality and effects caused by different reimbursement models, by using the inclusion criterion that the studies framed their questions in relation to the PHC choice reform. Furthermore, we did not include grey literature such as reports produced by government agencies or the regions, in which it is possible that additional findings may be presented (e.g. regarding costs and efficiency). It is also important to bear in mind that for some of the investigated topics only a few studies have been published. We cannot draw the conclusion that an effect does not exist based on a lack of evidence of such an effect in only a few studies. In addition, we did not assess the quality of the included articles and some of them are based on data from only one or a few regions while others cover all of Sweden. More studies are needed that can provide evidence on how the PHC choice reform has affected quality and costs. To be able to perform studies of high quality, we must know more about the length and content of PHC centre visits. The included studies have all looked at the number of visits as a measure but what is included in these visits is unclear.

## Conclusions

Summing up its first 15 years, we can conclude that the PHC choice reform has, to some extent, had the intended effects. Access and patient choice have been improved and there are now more private PHC centres. There are, however, indications of negative effects on equity, but it is still unclear how quality has been affected. Importantly, it seems as if the mechanisms behind the quality-enhancing function of the PHC reform do not function as intended. To improve the PHC choice system, the reimbursement models need constant oversight, and it is also important that there is a development of comprehensive clinical indicators that can be used when choosing providers and in the regions’ steering of the PHC providers. Furthermore, it must be discussed how a responsibility for population health can be integrated in PHC and the choice system.

## Supplemental Material

sj-docx-1-sjp-10.1177_14034948221095365 – Supplemental material for Fifteen years with patient choice and free establishment in Swedish primary healthcare: what do we know? Click here for additional data file. Supplemental material, sj-docx-1-sjp-10.1177_14034948221095365 for Fifteen years with patient choice and free establishment in Swedish primary healthcare: what do we know? by Mio Fredriksson and David Isaksson in Scandinavian Journal of Public Health
