# Supplementary material for: Fifteen years with patient choice and free establishment in Swedish primary healthcare: what do we know?
Source: Scand J Public Health. 2022 May 20;50(7):852–63. doi: 10.1177/14034948221095365 (PMC9578085; doi:10.1177/14034948221095365)
Supplement: sj-docx-1-sjp-10.1177_14034948221095365 – Supplemental material for Fifteen years with patient choice and free establishment in Swedish primary healthcare: what do we know? [file sj-docx-1-sjp-10.1177_14034948221095365.docx]

**Supplementary material. Description and classification of all included articles.**

| Title | Authors | Design | Geographical scope | Theme |
| --- | --- | --- | --- | --- |
| A decade of integration and collaboration: the development of integrated health care in Sweden 2000-2010 | Ahgren, Bengt; Axelsson, Runo | Qualitative | Sweden | Arguments about likely effects |
| Can pay-for-performance to primary care providers stimulate appropriate use of antibiotics? | Ellegard, Lina Maria; Dietrichson, Jens; Anell, Anders | Quantitative | Sweden | Governance and reimbursement systems |
| Can Private Provision of Primary Care Contribute to the Spread of Antibiotic Resistance? A Study of Antibiotic Prescription in Sweden | Granlund, David; Zykova, Yana V. | Quantitative | Västerbotten | Differences between private and public PHC centres |
| Changes in health care utilisation following a reform involving choice and privatisation in Swedish primary care: a five-year follow-up of GP-visits | Beckman, Anders; Anell, Anders | Quantitative | Skåne | Effects on access and equity |
| Choice and privatisation in Swedish primary care | Anell, Anders | Qualitative | Sweden | Governance and reimbursement systems |
| Choice of primary care provider: Results from a population survey in three Swedish counties | Glenngård, Anna H.; Anell, Anders; Beckman, Anders | Quantitative | 3 regions | Choice of PHC centre and use of information |
| Competition and integration in Swedish health care | Ahgren, Bengt | Qualitative | - | Arguments about likely effects |
| Competition, Capitation, and Coding: Do Public Primary Care Providers Respond to Increased Competition? | Dackehag, Margareta; Ellegard, Lina Maria | Qualitative | Skåne | Governance and reimbursement systems |
| Conditions and barriers for quality improvement work: a qualitative study of how professionals and health centre managers experience audit and feedback practices in Swedish primary care | Arvidsson, Eva; Dahlin, Sofia; Anell, Anders | Qualitative | 2 regions | Governance and reimbursement systems |
| Decision support systems for choosing a primary health care provider in Sweden | Ranerup, Agneta; Noren, Lars; Sparud-Lundin, Carina | Qualitative | 3 regions | Choice of PHC centre and use of information |
| Differences in unmet healthcare needs between public and private primary care providers: A population-based study | Lindstrom, Christine; Rosvall, Maria; Lindstrom, Martin | Quantitative | Skåne | Effects on access and equity |
| Does risk-adjusted payment influence primary care providers' decision on where to set up practices? | Anell, Anders; Dackehag, Margareta; Dietrichson, Jens | Quantitative | Sweden | Governance and reimbursement systems |
| Effects of pay-for-performance on prescription of hypertension drugs among public and private primary care providers in Sweden | Ellegard, Lina Maria | Quantitative | Sweden | Governance and reimbursement systems |
| Empowering the People: Public Responses to Welfare Policy Change | Bendz, Anna | Quantitative | West Sweden | Choice of PHC centre and use of information |
| Equity aspects of the Primary Health Care Choice Reform in Sweden - a scoping review | Burstrom, Bo; Burstrom, Kristina; Nilsson, Gunnar; Tomson, Goran; Whitehead, Margaret; Winblad, Ulrika | Review | Sweden | Effects on access and equity |
| Equity impact of a choice reform and change in reimbursement system in primary care in Stockholm County Council | Agerholm, Janne; Bruce, Daniel; de Leon, Antonio Ponce; Burstrom, Bo | Quantitative | Stockholm | Effects on access and equity |
| Free establishment of primary health care providers: effects on geographical equity | Isaksson, David; Blomqvist, Paula; Winblad, Ulrika | Quantitative | Sweden | Effects on access and equity |
| Governance, Government, and the Search for New Provider Models | Saltman, Richard; Duran, Antonio | Qualitative | Sweden | Governance and reimbursement systems |
| How do people choose to be informed? A survey of the information searched for in the choice of primary care provider in Sweden | Hoffstedt, Caroline; Fredriksson, Magnus; Winblad, Ulrika | Quantitative | Sweden | Choice of PHC centre and use of information |
| Increased registration of hypertension and cancer diagnoses after the introduction of a new reimbursement system | Hjerpe, Per; Bostrom, Kristina Bengtsson; Lindblad, Ulf; Merlo, Juan | Quantitative | Västra götaland | Governance and reimbursement systems |
| Information, switching costs, and consumer choice: Evidence from two randomised field experiments in Swedish primary health care | Anell, Anders; Dietrichson, Jens; Ellegard, Lina Maria; Kjellsson, Gustav | Quantitative | Skåne | Choice of PHC centre and use of information |
| Is choice of care compatible with integrated health care? An exploratory study in Sweden | Ahgren, Bengt; Nordgren, Lars | Qualitative | Southern Sweden | Choice of PHC centre and use of information |
| Is patient choice democratizing Swedish primary care? | Fredriksson, Mio | Qualitative | - | Governance and reimbursement systems |
| Is patient satisfaction in primary care dependent on structural and organizational characteristics among providers? Findings based on data from the national patient survey in Sweden | Glenngård, Anna H. | Quantitative | Sweden | Effects on quality |
| Is the quality of primary healthcare services influenced by the healthcare centre's type of ownership?-An observational study of patient perceived quality, prescription rates and follow-up routines in privately and publicly owned primary care centres | Maun, Andy; Wessman, Catrin; Sundvall, Par-Daniel; Thorn, Jorgen; Bjorkelund, Cecilia | Quantitative | Västra götaland | Differences between private and public PHC centres |
| Looking to Europe: Will Swedish healthcare reforms affect equity? | Burström, Bo | Qualitative | - | Arguments about likely effects |
| Market-oriented, demand-driven health care reforms and equity in health and health care utilization in Sweden | Burström, Bo | Qualitative | - | Arguments about likely effects |
| Market-orienting reforms in rural health care in Sweden: how can equity in access be preserved? | Kullberg, Linn; Blomqvist, Paula; Winblad, Ulrika | Qualitative | 3 regions | Governance and reimbursement systems |
| Money matters - primary care providers' perceptions of payment incentives | Vengberg, Sofie; Fredriksson, Mio; Burstrom, Bo; Burstrom, Kristina; Winblad, Ulrika | Qualitative | 2 regions | Governance and reimbursement systems |
| Neoliberal reforms in Swedish primary health care: For whom and for what purpose? | Dahlgren, Göran | Qualitative | - | Arguments about likely effects |
| Patient choice and provider competition – Quality enhancing drivers in primary care? | Vengberg, Sofie; Fredriksson, Mio; Winblad, Ulrika | Qualitative | 2 regions | Choice of PHC centre and use of information |
| Patient choice, entry, and the quality of primary care: Evidence from Swedish reforms | Dietrichson, Jens; Ellegard, Lina Maria; Kjellsson, Gustav | Quantitative | Sweden | Effects on quality |
| Patient choice, Internet based information sources, and perceptions of health care: Evidence from Sweden using survey data from 2010 and 2013 | Wahlstedt, Emma; Ekman, Bjorn | Quantitative | Skåne | Choice of PHC centre and use of information |
| Patients' perceptions of quality in Swedish primary care - a study of differences between private and public ownership | Andersson, Thomas; Eriksson, Nomie; Mullern, Tomas | Quantitative | Sweden | Differences between private and public PHC centres |
| Performing Through Privatization: An Ecological Natural Experiment of the Impact of the Swedish Free Choice Reform on Ambulatory Care Sensitive Conditions | Mosquera, Paola A.; San Sebastian, Miguel; Burstrom, Bo; Hurtig, Anna-Karin; Gustafsson, Per E. | Quantitative | Sweden | Effects on quality |
| Population preferences and choice of primary care models: A discrete choice experiment in Sweden | Hjelmgren, Jonas; Anell, Anders | Quantitative | Sweden | Choice of PHC centre and use of information |
| Preserving social equity in marketized primary care: strategies in Sweden | Winblad, Ulrika; Isaksson, David; Blomqvist, Paula | Qualitative | Sweden | Governance and reimbursement systems |
| Primary healthcare in transition - a qualitative study of how managers perceived a system change | Maun, Andy; Nilsson, Kerstin; Furaker, Carina; Thorn, Jorgen | Qualitative | Västra götaland | Governance and reimbursement systems |
| Productivity and patient satisfaction in primary care-Conflicting or compatible goals? | Glenngård, Anna H. | Quantitative | 2 regions | Governance and reimbursement systems |
| Promoting competition in Swedish primary care | Noren, Lars; Ranerup, Agneta | Mixed | 2 regions | Governance and reimbursement systems |
| Pursuing the objectives of support to providers and external accountability through enabling controls - a study of governance models in Swedish primary care | Glenngard, Anna Hager | Mixed | Sweden | Governance and reimbursement systems |
| Risk selection in primary care: a cross-sectional fixed effect analysis of Swedish individual data | Isaksson, David; Blomqvist, Paula; Pingel, Ronnie; Winblad, Ulrika | Quantitative | Sweden | Effects on access and equity |
| Short-term effects of a pay-for-performance programme for diabetes in a primary care setting: an observational study | Odesjo, H.; Anell, A.; Gudbjornsdottir, S.; Thorn, J.; Bjorck, S. | Quantitative | Västra götaland | Governance and reimbursement systems |
| Simply the best? The impact of quality on choice of primary healthcare provider in Sweden | Dahlgren, Cecilia; Dackehag, Margareta; Wändell, Per; Rehnberg, Clas | Quantitative | Stockholm | Choice of PHC centre and use of information |
| Socioeconomic distribution of GP visits following patient choice reform and differences in reimbursement models: Evidence from Sweden | Svereus, Sofia; Kjellsson, Gustav; Rehnberg, Clas | Quantitative | 3 regions | Effects on access and equity |
| Swedish citizens' opinions on decision support in primary healthcare | Ranerup A., Bendz A., Norén L. | Quantitative | Sweden | Choice of PHC centre and use of information |
| Tending to innovate in Swedish primary health care: a qualitative study | Avby, Gunilla; Kjellstrom, Sofia; Back, Monica Andersson | Qualitative | Jönköping | Governance and reimbursement systems |
| The Public–Private Pendulum — Patient Choice and Equity in Sweden | Anell, Anders | Qualitative | - | Governance and reimbursement systems |
| The trade-off between choice and equity: Swedish policymakers' arguments when introducing patient choice | Fredriksson, Mio; Blomqvist, Paula; Winblad, Ulrika | Qualitative | Sweden | Arguments about likely effects |
| To recommend the local primary health-care centre or not: what importance do patients attach to initial contact quality, staff continuity and responsive staff encounters? | Abrahamsson, Birgitta; Berg, Marie-Louise U.; Jutengren, Goran; Jonsson, Annikki | Quantitative | Västra götaland | Choice of PHC centre and use of information |
| Weak association between socioeconomic Care Need Index and primary care visits per registered patient in three Swedish regions | Anell, Anders; Dackehag, Margareta; Ellegard, Lina Maria | Quantitative | 3 regions | Governance and reimbursement systems |
| When do people choose to be informed? Predictors of information-seeking in the choice of primary care provider in Sweden | Hoffstedt, Caroline; Fredriksson, Magnus; Lenhoff, Hakan; Winblad, Ulrika | Quantitative | Sweden | Choice of PHC centre and use of information |
